# First Diagonal to Left Ventricle Fistula With LAD Collateral Flow: An Unusual Coronary Detour

**DOI:** 10.1016/j.jaccas.2026.109518

**Published:** 2026-07-29

**Authors:** Johnathan Stephan, Mohammed Uddin, Everett Woods, Marcel Zughaib

**Affiliations:** Department of Cardiology, Henry Ford Providence Hospital, Bloomfield Hills, Michigan, USA

**Keywords:** chronic total occlusion, coronary artery anomaly, coronary artery fistula, coronary cameral fistula, coronary collateral circulation, coronary steal, left anterior descending artery, percutaneous coronary intervention

## Abstract

**Background:**

Coronary artery fistulas are rare vascular anomalies connecting a coronary artery to a cardiac chamber or vessel. While typically associated with ischemia via coronary steal, they can paradoxically serve a protective role.

**Case Summary:**

We present a 57-year-old Asian male with 2 weeks of effort angina who was found to have an elevated high-sensitivity troponin. Urgent coronary angiography revealed a 100% mid left anterior descending artery (LAD) occlusion with collateral flow supplied predominantly by a fistulizing first diagonal artery (D1) communicating with the left ventricle.

**Discussion:**

The D1 fistula branches provided critical left-to-left collateral perfusion to the occluded LAD territory, likely limiting ischemic burden and preserving ventricular function. The LAD was successfully recanalized with drug-eluting stent placement, and percutaneous transluminal coronary angioplasty of the D1 was performed.

**Take-Home Message:**

This case demonstrates that coronary artery fistulas are not inherently pathological and highlights the importance of individualized anatomical assessment when planning coronary interventions.

## Visual Summary

**Figure:**
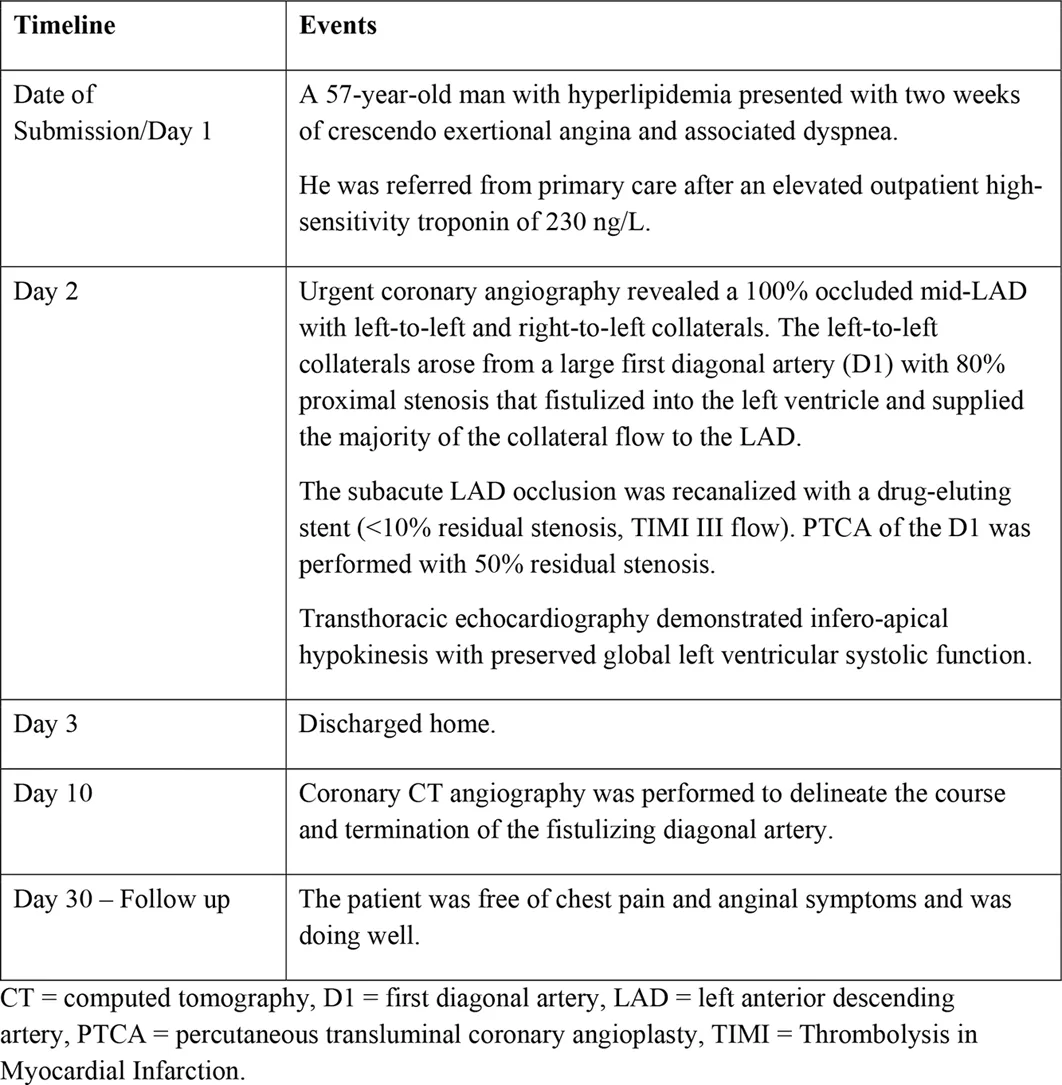

## History of Presentation

A 57-year-old Asian male with history of hyperlipidemia presented with a 2-week history of effort angina with associated dyspnea. The chest pain was described as substernal pressure, nonradiating, and precipitated with exertion. Symptoms began abruptly over the weekend and progressively worsened limiting daily activities. He denied syncope, palpitations, orthopnea, or lower extremity edema.

**Take-Home Message**

• This case illustrates that coronary artery fistulas are not inherently pathological and highlights the importance of individualized anatomical assessment when planning coronary interventions.

He initially presented to his primary care physician and was found to have an elevated outpatient high-sensitivity troponin level of 230 ng/L. This prompted urgent referral to cardiology for further evaluation.

## Past Medical History

The patient had a history of hyperlipidemia; there was no prior history of coronary artery disease or cardiac intervention.

## Differential Diagnosis

Given the presentation of subacute exertional angina with troponin elevation, the differential diagnosis included non–ST-segment elevation myocardial infarction, subacute total occlusion of the left anterior descending artery (LAD) with collateralization, demand ischemia, myocarditis, coronary artery anomaly with malignant course, and coronary artery fistula with steal physiology.

## Investigations

Initial electrocardiogram demonstrated normal sinus rhythm with nonspecific ST-segment changes without ST-segment elevation. High-sensitivity troponin was elevated at 230 ng/L. Transthoracic echocardiography revealed inferoapical hypokinesis with preserved overall left ventricular systolic function.

**Figure 1:**
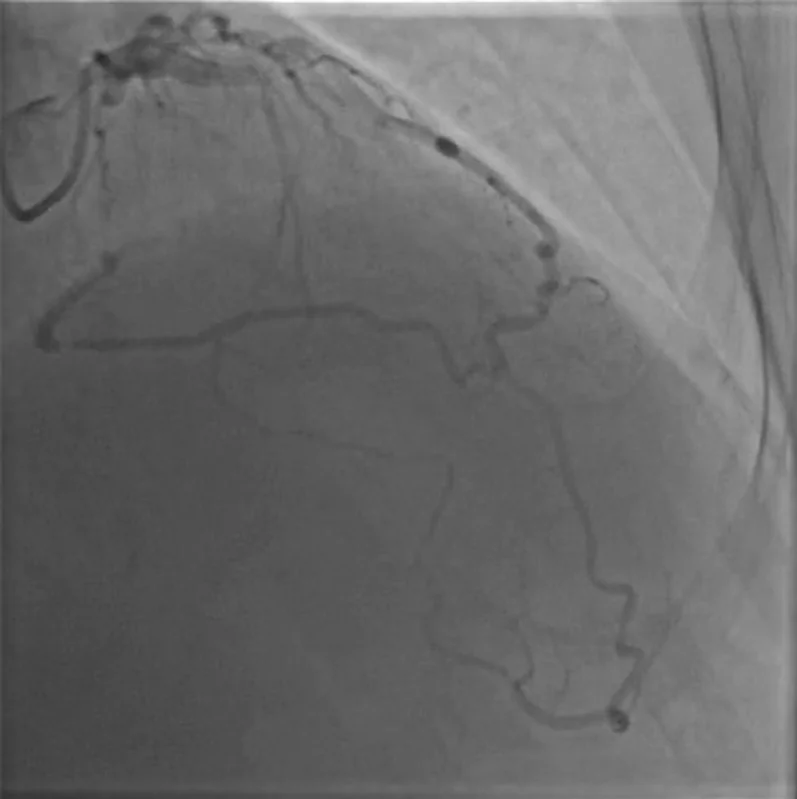
Invasive Coronary Angiography Left anterior descending artery with subacute total occlusion with collateral flow from first diagonal artery, which is fistulizing to the left ventricle.

Urgent invasive coronary angiography demonstrated 100% occluded mid-LAD with left-to-left and right-to-left collaterals and first diagonal artery (D1) with 80% proximal stenosis with fistulization into the left ventricle. The left-to-left collaterals branch from the fistulizing D1 provided the majority of the collateral flow to the LAD (Video 1, Figure 1).

**Figure 2:**
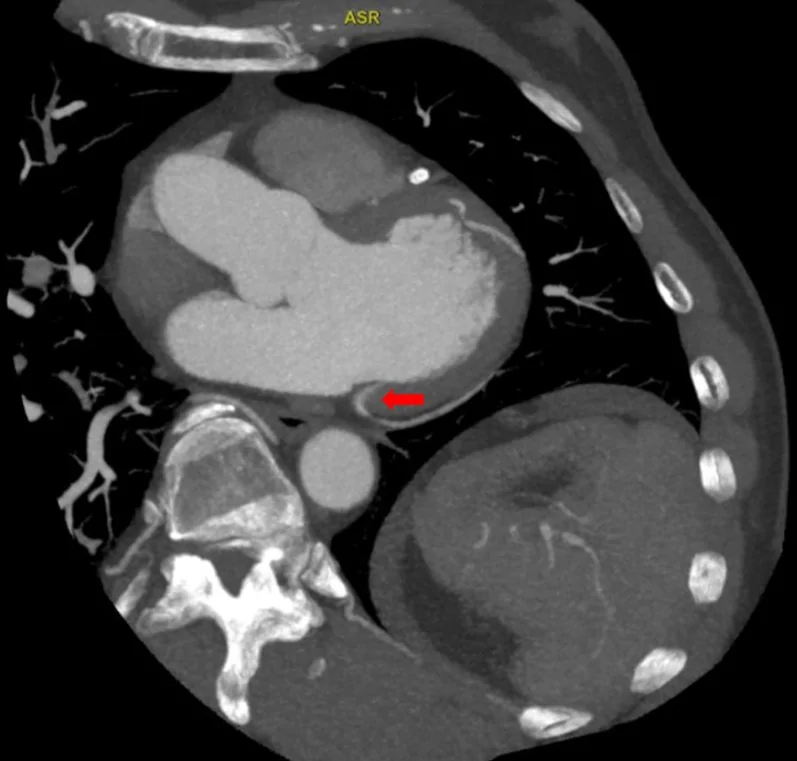
Coronary Computed Tomography Angiography The first diagonal artery fistula inserting into the basal aspect of the left ventricle.

**Figure 3:**
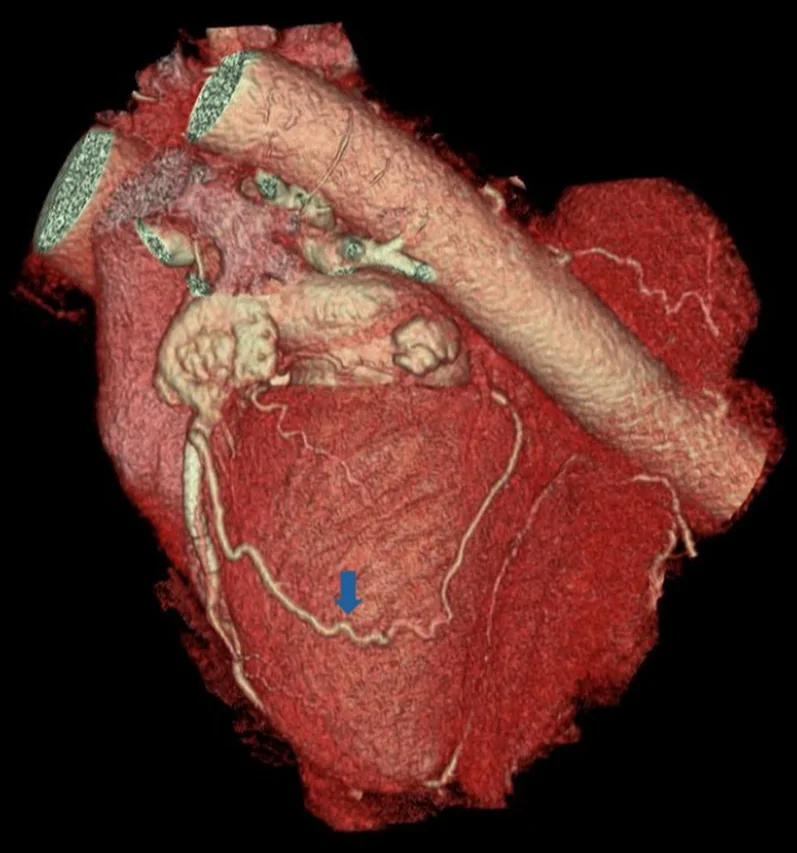
Coronary Computed Tomography Angiography Volume Rendering First diagonal artery traversing the left ventricular free wall and inserting into the basal aspect of the lateral wall.

Coronary computed tomography angiography identified an anomalous communication between the D1 and the left ventricular cavity, consistent with a coronary cameral fistula (Figure 2). Three-dimensional volume rendering demonstrated the D1 with abnormal course across the lateral heart and toward the atrioventricular groove, where it fistulized into the left ventricle (Figure 3).

## Management

**Figure 4:**
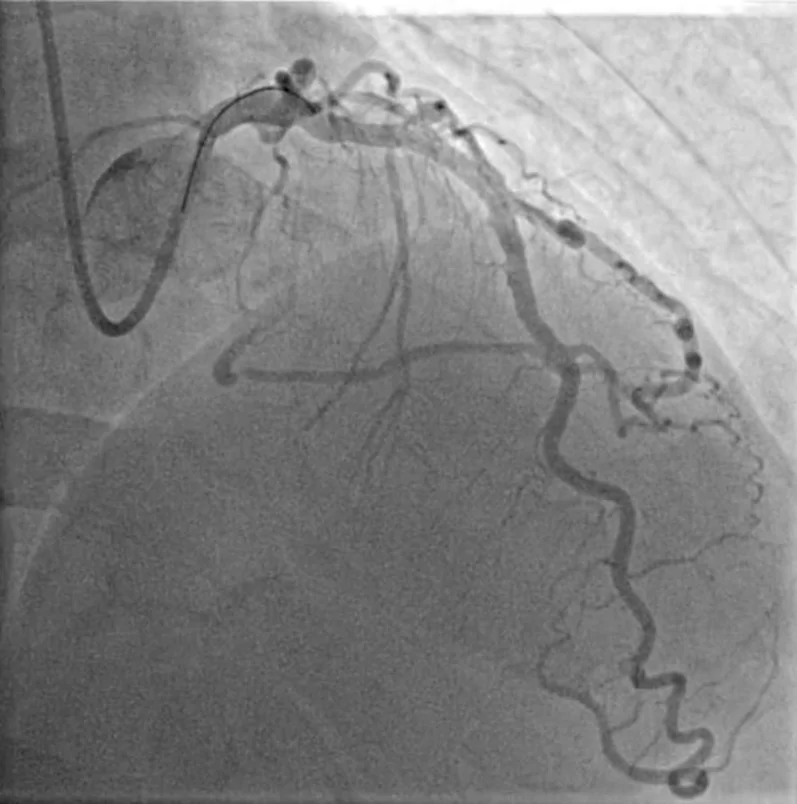
Invasive Coronary Angiography Showing Revascularized LAD LAD = left anterior descending artery.

Given ongoing symptoms and biomarker elevation consistent with a subacute myocardial infarction, percutaneous coronary intervention was pursued. The mid-LAD subacute total occlusion was successfully recanalized with drug-eluting stent implantation, achieving <10% residual stenosis and restoration of antegrade flow. Percutaneous transluminal coronary angioplasty of the proximal D1 lesion was performed, with improvement to 50% residual stenosis (Video 2, Figure 4). The patient was asymptomatic following revascularization.

Fistula closure was deferred because there was no significant volume overload, no evidence of ischemia attributable to steal physiology, and because restoration of antegrade LAD flow reduced reliance on collateral circulation. The patient was initiated on guideline-directed medical therapy including dual antiplatelet therapy and high-intensity statin.

## Outcome and Follow-Up

The patient's exertional angina resolved following revascularization. He was discharged on dual antiplatelet therapy, a high-intensity statin, and risk factor modification counseling. At follow-up, he remained symptom free without recurrent angina, and repeat imaging confirmed preserved left ventricular systolic function.

## Discussion

Previously, coronary artery anomalies were estimated to have a prevalence of 0.3% to 1.3% in diagnostic coronary angiography, with coronary artery fistulas (CAFs) accounting for 0.2% to 0.4% of congenital cardiac anomalies.^1^ The prevalence of CAFs was found to be closer to 0.9% with the improved detection provided by coronary computed tomography angiography; coronary artery–to–cardiac chamber fistulas comprised only 8.9% of these cases.^2^ The majority of CAFs drain into right-sided low-pressure structures such as the right ventricle, right atrium, or pulmonary artery. Left ventricular coronary cameral fistulas are exceedingly rare, accounting for only 3% of all CAF cases.^3^^,^^4^ The primary pathophysiology associated with CAFs is the coronary steal phenomenon. This is driven by flow diverting from the coronary tree into a low-resistance receiving cavity through a diastolic pressure gradient, leading to ischemia in the myocardium distal to the fistula origin.^5^

The present case represents a paradoxical scenario. Rather than functioning as a source of coronary steal, the fistulizing D1 appeared to serve as a conduit for protective collateral perfusion to the occluded LAD. The presence of a subacute total occlusion almost certainly drove the progressive recruitment of collateral channels from D1. This response is consistent with the adaptive remodeling of the coronary microcirculation in the setting of epicardial occlusion. In the presence of total occlusions, preformed coronary collaterals are recruited and undergo remodeling to provide an alternative source of myocardial blood flow, mitigating ischemic injury.^6^

We concluded that the LAD lesion was subacute and was the culprit of the patient's presentation based on 3 main factors. First, the timing of the patient's crescendo angina with the concurrent troponin elevation is consistent with an acute coronary syndrome. Second, there was no significant disease in the right coronary or left circumflex arteries to account for the clinical picture. Third, the lesion was easily crossed with a workhorse Runthrough wire rather than requiring dedicated chronic total occlusion wires, behaving more consistently with a subacute lesion rather than long-standing chronic total occlusion. This distinction is clinically relevant, as the subacute time course allowed sufficient time for collateral recruitment, which preserved perfusion to the LAD territory and prevented a likely ST-segment elevation myocardial infarction presentation despite complete occlusion.

A closely analogous scenario was reported by Yi et al,^7^ who described a left circumflex coronary artery–to–pulmonary artery fistula that served as the exclusive collateral source to a proximally occluded LAD. After successful recanalization of the LAD, the collateral channels originating from the fistula promptly disappeared.^7^ Our case parallels this physiology, though notably distinct in that the fistula drained into the left ventricle rather than the pulmonary artery, and the collateral function was mediated through D1 rather than the circumflex. The combination of a coronary cameral fistula with coexistent significant coronary artery disease and a subacute total occlusion is itself rare, having been described in only isolated case reports.

The decision to improve flow through D1 to the fistula via percutaneous transluminal coronary angioplasty, rather than closure of the vessel, was reached on physiologic grounds. Given the vessel's moderate size with both ostial stenosis and distal stenosis, immediately preceding to the left ventricular fistula, we suspected the amount of myocardium at risk was high. Furthermore, the absence of volume overload, steal-related ischemia, or other conventional indications supported a conservative approach to the fistula itself. In our patient, the successful restoration of antegrade flow in the LAD effectively reduced the hemodynamic reliance on the D1 collateral circuit. This approach is consistent with the principle that small, asymptomatic fistulas without hemodynamic consequence are appropriately managed conservatively.^8^

This case contributes to the limited but growing literature documenting protective or adaptive roles for coronary fistulas in the setting of obstructive coronary artery disease, and it illustrates that the hemodynamic significance of a fistula cannot be determined in isolation; it must be interpreted within the full angiographic and clinical context.

## Conclusions

We describe a rare first diagonal artery–to–left ventricle coronary cameral fistula that functioned as a protective collateral source sustaining perfusion to an occluded LAD. While CAFs are conventionally associated with coronary steal and ischemia, this case illustrates that their hemodynamic impact is highly context dependent. When a fistula exists in the setting of an epicardial occlusion, it may paradoxically serve an adaptive rather than deleterious role. Individualized anatomical and functional assessment is essential when planning revascularization in patients with complex coronary anomalies, and fistula closure should not be undertaken reflexively without careful consideration of the collateral physiology.

## Funding Support and Author Disclosures

The authors have reported that they have no relationships relevant to the contents of this paper to disclose.

## Appendix

Video 1 Invasive Coronary Angiography LAD with subacute total occlusion with collateral flow from first diagonal artery, which is fistulizing to left ventricle.

Video 2 Invasive Coronary Angiography Revascularized LAD.
